# Dysphagia as a manifestation of esophageal tuberculosis: a report of two cases

**DOI:** 10.1186/1752-1947-5-447

**Published:** 2011-09-08

**Authors:** Joana Gomes, Ana Antunes, Aurora Carvalho, Raquel Duarte

**Affiliations:** 1 Centro Hospitalar de Gaia/Espinho, EPE, Rua Conceição Fernandes 4434-502 Vila Nova de Gaia, Portugal

## Abstract

**Introduction:**

Esophageal involvement by *Mycobacterium tuberculosis *is rare and the diagnosis is frequently made by means of an esophageal biopsy during the evaluation of dysphagia. There are few cases reported in the literature.

**Case presentation:**

We present two cases of esophageal tuberculosis in 85- and 65-year-old male Caucasian patients with initial complaints of dysphagia and epigastric pain. Upper gastrointestinal endoscopy resulted in the diagnosis of esophageal tuberculosis following the biopsy of lesions of irregular mucosa in one case and a sessile polyp in the other. Pulmonary tuberculosis was detected in one patient. In one patient esophageal stricture developed as a complication. Antituberculous therapy was curative in both patients.

**Conclusion:**

Although rare, esophageal tuberculosis has to be kept in mind in the differential diagnosis of dysphagia. Pulmonary involvement has important implications for contact screening.

## Introduction

Tuberculosis of the esophagus is a rare condition, even in countries with a high incidence of tuberculosis (TB) [1,2], and studies estimate that it constitutes about 0.3% of gastrointestinal TB cases [3]. Involvement of the gastrointestinal tract occurs through ingestion of infected sputum or hematogenous spread from primary pulmonary TB [4]. Most cases of esophageal tuberculosis are secondary to direct extension from adjacent structures, such as mediastinal lymph nodes or pulmonary sites. Primary esophageal tuberculosis is even rarer [5]. Esophagic involvement by tuberculosis usually affects the middle third of the esophagus at the carina level [6]. The most common symptoms are dysphagia or retrosternal pain, but odynophagia and weight loss may also be present.

We present two case reports of esophageal involvement by *Mycobacterium tuberculosis *infection in immunocompetent persons.

## Case presentations

### Case one

**Figure 1:**
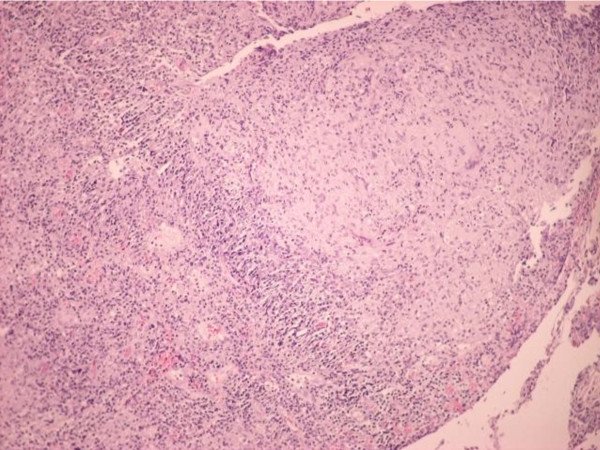
**Histology of esophageal mucosa in case one**. Esophageal mucosa with extensive ulceration and inflammatory lesion with epithelioid granulomas. The acid-fast staining was positive.

A Portuguese Caucasian man, 89 years old, with a history of hypertension and benign prostatic hypertrophy, started to experience dysphagia, epigastric pain and anorexia one month prior to presentation. He was treated with omeprazole and sucralfate without any improvement. He had a normal blood count, with an erythrocyte sedimentation rate of 16 mm (normal range: 1-7 mm). An upper gastrointestinal endoscopy was performed. This revealed congestion of the entire esophageal mucosa, mainly in the proximal portion (20-25 cm from incisors), with easy bleeding to the touch, some irregular mucosa (biopsy one performed) and, in the lower part of the esophagus, an irregular mucosa with nodular areas (biopsy two performed). Diagnostic hypotheses were esophageal cancer and esophagitis. The histological examination for biopsy one revealed heavy lymphocytic infiltrate and polymorphonuclear cells, with ulceration, without lesions of malignancy. The histological examinations of biopsy two showed esophageal mucosa with extensive ulceration, inflammatory lesions with epithelioid granulomas, and acid-fast alcohol resistant microorganisms on staining (Figure 1). A chest radiography showed no lesions. A purified protein derivative skin test was negative. Because of persistent cough, mycobacteriological sputum examination was performed; the smear was negative but the culture was positive on the second month. Human immunodeficiency virus (HIV) -1 and -2 serology was negative.

**Figure 2:**
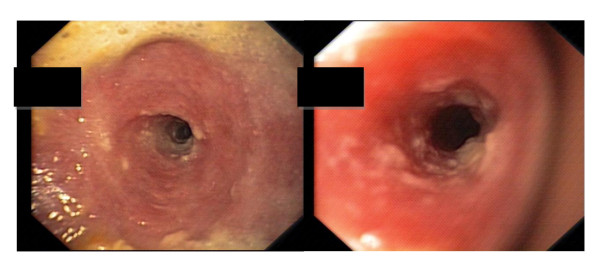
**Upper gastrointestinal endoscopy after starting treatment in case one revealed cicatricial stenosis of the esophagus**.

Our patient began antituberculous therapy with isoniazid, rifampicin, pyrazinamide and ethambutol with symptomatic improvement. The persistence of dysphagia to solids led to upper gastrointestinal endoscopy repetition three months after starting antituberculous treatment, and revealed cicatricial stenosis of his esophagus requiring repeated esophageal dilatations (Figure 2). He completed treatment with two months of isoniazid, rifampicin, pyrazinamide and ethambutol, followed by four additional months of rifampicin and isoniazid. No further esophageal dilatations were required and our patient has no gastrointestinal complaints.

### Case two

**Figure 3:**
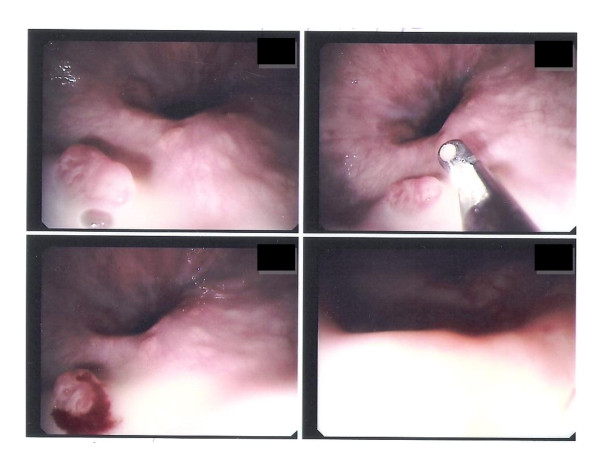
**Upper gastrointestinal endoscopy in case two**. Sessile polyp with irregular surface in the distal third of the esophagus.

The second patient was a Portuguese Caucasian man, 65 years old, with a history of hypertension, Ménière's syndrome and a known allergy to penicillin. He reported anorexia and weight loss of 8 kg two years earlier. The examination performed at that time revealed a right pleural effusion that resolved spontaneously over six months of follow-up, with no etiological diagnosis made. He presented with epigastric and left upper abdominal quadrant pain and had an erythrocyte sedimentation rate of 15 mm and a C-reactive protein level of 5.8 mg/dL (normal range: < 0.5 mg/dL). An abdominal ultrasonography showed mild polypoid thickening in his gallbladder without calculus, with no pain to elective area compression and no other changes. An upper gastrointestinal endoscopy revealed a sessile 6 mm polyp with an irregular surface in the distal third of his esophagus, located 2 cm above the junction with his stomach (biopsy performed, see Figure 3), which was removed through endoscopy. Histology revealed the presence of epithelioid granulomas with multinucleated giant Langhans cells, caseous necrosis and acid-fast bacilli. A chest radiography showed no relevant changes. A purified protein derivative skin test was positive after 48 hours.

**Figure 4:**
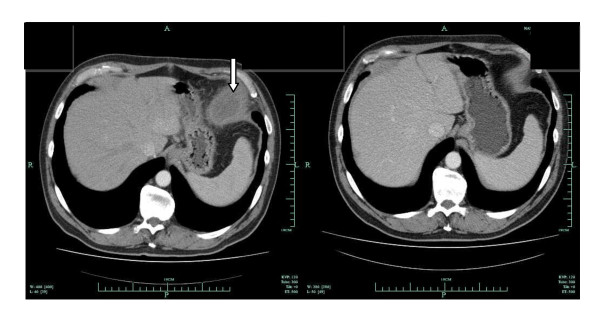
**Thoracic computed tomography scan in case two showing a left juxtadiaphragmatic effusion (arrow) which almost disappeared after two weeks of antituberculous therapy**.

Our patient started therapy with isoniazid, rifampicin, pyrazinamide and ethambutol. Serology for HIV1 and 2 was negative. Because of worsening pain in his left upper abdomen irradiating to the left thoracic region, a computed tomography scan was done and revealed a left juxtadiaphragmatic fluid collection. After two weeks of antituberculous therapy, there was a significant reduction of the effusion (Figure 4). Our patient completed treatment with four drugs during the first two months and an additional four months of therapy with isoniazid and rifampicin. The clinical outcome was good. No drug toxicity or complications were observed.

## Discussion

The diagnosis of esophageal tuberculosis is rare, hence there is a need for a high clinical suspicion. Tuberculosis rarely causes dysphagia, which occurs due to esophageal ulcers, tracheoesophageal fistula or extrinsic compression by the mediastinal or neck lymph nodes [6,7]. Tuberculosis can involve the esophagus as a primary infection or as a secondary manifestation of disease reactivation [8]. Case one was clearly a case of secondary esophageal tuberculosis, as there was proven pulmonary tuberculosis by sputum mycobacteriological examination. However, in case two there was a past history of pleural effusion that resolved spontaneously, as well as a left juxtadiaphragmatic effusion that resolved with antituberculous therapy and was most likely caused by *M. tuberculosis*. Given these facts, we cannot state for sure what was the primary focus of infection.

The most frequent symptom reported in esophageal tuberculosis is dysphagia, which occurs in about 90% of cases [2,9]. Other symptoms are odynophagia and retrosternal pain and the occurrence of symptoms such as fever, weight loss and anorexia [2,9] is also common. In our cases, dysphagia and epigastric pain were the cardinal symptoms.

Esophageal tuberculosis lesions can involve any segment of this organ, but is most often located in the middle third of the esophagus because of its proximity to the hilar and mediastinal lymph nodes surrounding the bifurcation of the trachea [2,9,10]. In the cases presented, esophageal involvement was at the distal level. The most common macroscopic finding is an esophageal ulcer as observed in case one. However, hypertrophic growth as esophageal polyps may also be present as in case two [5,11]. Esophageal carcinoma is part of the differential diagnosis as was the case for both our patients. Diagnosis is usually made by upper gastrointestinal endoscopy with histology examination showing epithelioid granuloma with Langhans cells, central necrosis and acid-fast bacilli. This was the method that allowed the diagnosis in both cases. In secondary esophageal tuberculosis, diagnosis may be suggested by confirmation of tuberculosis involving adjacent structures [5], which was not possible in case two, given the quick resolution of the abdominal effusion after starting antituberculous therapy.

Esophageal tuberculosis treatment is based on chemotherapy with four drugs (isoniazid, rifampicin, pyrazinamide and ethambutol) in a first phase lasting for two months, followed by a period of four to six months with two drugs (isoniazid and rifampicin). There are cases where treatment was successfully carried out with only three drugs for six months, excluding ethambutol [12,13]. Surgical treatment is reserved for complications such as esophageal, tracheoesophageal and aortoesophageal fistulas, the latter of which can lead to death by massive hematemesis [14]. In both cases presented, six-months of antituberculous therapy was curative.

Esophageal strictures may result from external compression of the esophagus due to mediastinal or cervical lymph nodes as well as mediastinal fibrosis induced by tuberculosis. This condition results in long and narrow strictures that are difficult to dilate, and in which dilation may be associated with a higher rate of complications [15].

Esophageal stenosis as a complication of esophageal tuberculosis is rare and there are few reports in the literature [16,17]. The stenosis in case one was probably the result of the healing process, but was severe and repeated esophageal dilatation was needed to maintain esophagogastric transit.

## Conclusion

Although rare, esophageal tuberculosis must be kept in mind in patients with dysphagia, especially in countries with high prevalence of tuberculosis, even in immunocompetent patients. Active pulmonary tuberculosis should be ruled out, since early recognition of this infection is very important for public health. Treatment of esophageal tuberculosis with antituberculous drugs is curative, although complications may sometimes occur as in our case. Finally, esophageal cancer must be included in the differential diagnosis of the endoscopic findings in this situation.

## Consent

Written informed consent was obtained from both patients for publication of this case series and accompanying images. A copy of the written consent is available for review by the Editor-in-Chief of this journal.

## Competing interests

The authors declare that they have no competing interests.

## Authors' contributions

JG was a major contributor in writing and revising the manuscript. RD was involved in drafting the manuscript and revising it critically for important intellectual content. AC and AA analyzed and interpreted the patient data regarding mycobacteriological examination. All authors were responsible for the diagnosis, treatment and follow-up of the patients whose case reports were described. All authors read and approved the final manuscript.
